# Characterization of the Bacterial Community Associated with Methane and Odor in a Pilot-Scale Landfill Biocover under Moderately Thermophilic Conditions

**DOI:** 10.4014/jmb.2103.03005

**Published:** 2021-04-21

**Authors:** Hyoju Yang, Hyekyeng Jung, Kyungcheol Oh, Jun-Min Jeon, Kyung-Suk Cho

**Affiliations:** 1 Department of Environmental Science and Engineering, Ewha Womans University, Seoul 03760, Republic of Korea; 2 Green Environmental Complex Center, Suncheon 57992, Republic of Korea

**Keywords:** Methane, odor, thermophile, thermotolerance, bacterial community, biological system

## Abstract

A pilot-scale biocover was constructed at a sanitary landfill and the mitigation of methane and odor compounds was compared between the summer and non-summer seasons. The average inlet methane concentrations were 22.0%, 16.3%, and 31.3%, and the outlet concentrations were 0.1%, 0.1%, and 0.2% during winter, spring, and summer, respectively. The odor removal efficiency was 98.0% during summer, compared to 96.6% and 99.6% during winter and spring, respectively. No deterioration in methane and odor removal performance was observed even when the internal temperature of the biocover increased to more than 40°C at midday during summer. During summer, the packing material simultaneously degraded methane and dimethyl sulfide (DMS) under both moderately thermophilic (40–50°C) and mesophilic conditions (30°C). *Hyphomicrobium* and *Brevibacillus*, which can degrade methane and DMS at 40°C and 50°C, were isolated. The diversity of the bacterial community in the biocover during summer did not decrease significantly compared to other seasons. The thermophilic environment of the biocover during summer promoted the growth of thermotolerant and thermophilic bacterial populations. In particular, the major methane-oxidizing species were *Methylocaldum* spp. during summer and *Methylobacter* spp. during the nonsummer seasons. The performance of the biocover remained stable under moderately thermophilic conditions due to the replacement of the main species and the maintenance of bacterial diversity. The information obtained in this study could be used to design biological processes for methane and odor removal during summer and/or in subtropical countries.

## Introduction

Methane is a significant greenhouse gas that has a 28–36 times stronger effect on global warming than carbon dioxide [1]. Landfills are a major source of methane due to the anaerobic digestion of organic matter [2], during which odor compounds, including hydrogen sulfide (H_2_S), methanethiol, dimethyl sulfide (DMS), and volatile organic compounds, are also produced as byproducts [3-5]. Odor compounds are not only a nuisance but also pose a potential hazard to human health at high concentrations, with long-term exposure often leading to emotional stress and physical symptoms such as anxiety, headaches, vomiting, eye irritation, and respiratory problems [6-9]. Therefore, the mitigation of both methane and odor is necessary for anaerobic digestion processes.

Methane and odor compounds emitted by anaerobic digestion can be mitigated using biological, chemical, or physical treatment [3, 10-12]. Biological treatment is more environmentally friendly and safer than chemical and physical approaches because it does not require the use of chemicals and can be conducted at normal temperatures and pressures [3, 10, 11]. Biological treatment also offers high treatment efficiency, simple systems, and low treatment costs [3, 12]. Therefore, biological systems such as biocovers and biowindows have attracted attention as promising technologies for the control of the methane and odor compounds emitted from landfills [10, 11].

Microbial activity, which determines the performance of biological treatment systems [13], is primarily influenced by environmental factors such as temperature, pH, and moisture content [3, 14]. For example, if the temperature of the biological treatment system is outside the range that most microorganisms can tolerate, the microbial activity is dramatically lower [14]. Most biological systems designed to mitigate the methane and odor compounds emitted by landfills employ mesophilic microorganisms [15-17]. Because the activity of mesophilic microbes decreases rapidly at low temperatures, it could be expected that the performance of biological treatment systems would deteriorate during winter. However, this is not the case because the internal temperature of these systems is maintained at around 8–18°C due to the heat generated by the biodegradation of methane and odor compounds and the effect of insulating materials [10, 11, 18].

Mesophilic microorganisms are generally less active under moderately thermophilic conditions of 40°C or more. At midday during summer, the temperature inside biological treatment systems may rise to over 40°C due to the effects of solar radiation. Information on the composition and dynamics of the microbial community under moderately thermophilic conditions is thus essential when designing and setting the operating parameters for biological treatment systems during the hot season or in subtropical countries, but there is little information available on this. Therefore, in this study, the methane and odor removal performance of a pilot-scale biocover during the summer and non-summer seasons was compared. In particular, the structure of the bacterial community during summer was characterized and compared with the non-summer seasons. In addition, the degradation of methane and odor compounds by the packing material sampled from the biocover during summer was evaluated under moderately thermophilic conditions. Thermotolerant or thermophilic methane- and odor-degrading bacteria were also isolated and their roles in the degradation of methane and odor compounds investigated.

## Materials and Methods

### Packing Material for the Biocover

A pilot-scale biocover was constructed at the Gwangyang Sanitary Landfill, located at latitude 34°58’0’’ and longitude 127°38’35’’. This sanitary landfill began operation in 1996 and has a total disposal capacity of 3,145,291 m^3^ [10, 19]. The packing material for the biocover was a mixture of soil, perlite (Kyungdong One Co. Ltd., Korea), food waste compost, and earthworm cast (Kumhosilup, Korea). The physiochemical characteristics of these materials were described previously by Lee *et al*. [10]. The soil, which had been used as an interim landfill cover, was provided by the landfill facility, and the food waste compost was acquired from the Gwangyang Food Waste Resource Facility (Korea). Perlite was used to increase the gas permeability of the biocover [10, 11]. The compost and earthworm cast were used as bacterial sources for the simultaneous removal of methane and odor compounds [10, 11]. Based on preliminary testing, the ratio of perlite, soil, food waste compost, and earthworm cast was set at 2.5:2.5:0.6:0.6 (v/v). The initial moisture and organic content of the mixture were 16.44 ± 0.13% and 5.67 ± 0.02%, respectively.

### Construction of the Pilot-Scale Biocover and Packing Material

A schematic diagram of the biocover (2.5 m wide × 2.5 m long × 1.2 m deep) is presented in Fig. S1. For thermal insulation, polystyrene foam boards (5 cm thick) were placed along the inside of the walls. The solid waste was first covered with a 0.23-m thick layer of gravel (particle diameter of 2–5 cm) and then a polypropylene non-woven textile sheet (Kyungdong One Co. Ltd.,). A 0.9-m-thick soil layer was placed on top of the textile cover. A perforated pipeline was installed at the bottom of the biocover and connected to polyvinyl chloride (PVC) pipes for biocover inlet gas sampling. An acrylic chamber (2.5 m wide × 2.5 m long × 0.3 m high) was installed on the surface of the biocover for gas sampling from the biocover surface (*i.e*., biocover outlet gas sampling). Biocover performance was monitored for 260 days from December 2016 to August 2017.

### Ambient Temperature, Precipitation, and Physicochemical Properties of the Packing Material

Ambient temperature and precipitation measurements during the experimental period were obtained from the Gwangyang Automatic Weather Station operated by the Korea Meteorological Administration. The packing material in the biocover was sampled between 11 and 12 o’clock on days 0, 40, 68, 99, 133, 163, 198, 238, and 252 at 0–15 cm (upper layer), 15–30 cm (middle layer), and 30–50 cm (bottom layer) from the surface of the biocover. Immediately before taking the sample, the temperature of the packing material in each layer was measured using a portable digital thermometer (SDT200, Summit Co. Ltd., Korea).

After being passed through a 2-mm sieve, the samples were stored at 4°C for the assessment of their physicochemical properties (pH, moisture content, and organic matter content) and at –20°C for the analysis of the bacterial community. The moisture and organic matter content of the samples were measured based on the Korean Standard Soil Analysis Method [10] and the Korean Standard Waste Analysis Method [10], respectively. To measure the pH of the samples, 3 g of each sample was mixed with 20 mL of distilled water, and the supernatant was collected after allowing the particles to settle for 5 min. The pH of the resulting supernatant was measured with a pH meter (Thermo Orion 535A, USA).

### Gas Analysis

Gas samples from the inlet port and the surface of the biocover were collected on days 12, 39, 63, 98, 124, 165, 182, 223, and 253. The gas sampling was conducted using the same method described in a previous study [10]. The methane concentration in the gas samples was measured using a gas chromatograph equipped with a flame ionization detector [10]. The methane levels were also measured in the field using a biogas check analyzer (Geotechnical Instruments, UK) [10]. The concentrations of 22 odor compounds designated as key offensive odors by the Korean Odor Prevention Law were analyzed using the same methods described in a previous study [10]. Complex odor compounds were analyzed using the odor dilution ratio (ODR) [20]. Details on the calculation of the ODR are available in Lee *et al*. [10]. The removal efficiency of the methane and complex odor compounds was calculated as the difference in the concentration of the target compounds between the inlet and the surface of the biocover.

### Bacterial Community Analysis Using Illumina MiSeq Sequencing

The packing material sampled at 15–30 cm on days 0–252 was used to characterize the bacterial community dynamics in the biocover. For DNA extraction, 0.5 g of each sample was transferred to a microtube from the stored bottle, and the DNA was extracted using a NucleoSpin Soil Kit (Macherey-Nagel GmbH, Germany) and a Mini-BeadBeater-8 system (BioSpec, USA). DNA extraction was performed following the manufacturer’s instructions. The extracted DNA samples were eluted with 50 μl of an elution buffer and stored at –20°C before analysis. The extracted DNA was used as a PCR template to analyze the bacterial community with an Illumina MiSeq sequencing platform (Macrogen Inc., Korea) using the same method described in our previous paper [11]. Each composite primer was designed based on 515f and 806r primers [21]. Sequences shorter or longer than the target sequence were cut using CD-HIT-OTU [22], and chimera and noise were eliminated. The sequences with over 97% similarity were classified into operational taxonomic units (OTUs). Using the UCLUST algorithm [23], the taxonomy for each OTU was assigned based on the 16S rRNA RDP database. The Chao1 richness estimator and the Shannon index were also calculated [24]. The obtained sequence data were deposited into the National Center for Biotechnology Information (NCBI) Sequence Read Archive (https://www.ncbi.nlm.nih.gov/) under accession number SRP185598. Bacterial community dynamics were analyzed using principal component analysis (PCA) with UniFrac [25] and CANOCO 4.5 software (Microcomputer Power, USA).

### Simultaneous Removal of Methane and Dimethyl Sulfide by the Packing Materials under Moderately Thermophilic Conditions

The simultaneous removal of methane and odor compounds was evaluated at 40°C and 50°C for three packing material samples taken on day 252 at 0–15 cm, 15–30 cm, and 30–50 cm from the surface of the biocover. In order to compare the removal efficiency under moderately thermophilic and medium temperature conditions, the same experiment was carried out at 30°Cs. DMS was selected as a representative odor compound. Five grams of the wet samples and 20 ml of nitrated mineral salt (NMS) medium were added to 600-ml serum bottles. The NMS medium contained 1.0 g/l MgSO_4_·7H_2_O, 0.2 g/l CaCl_2_·6H_2_O, 1.0 g/l KNO_3_, 0.272 g/l KH_2_PO_4_, and 0.717 g/l Na_2_HPO_4_·12H_2_O. The serum bottles were sealed with a butyl rubber stopper, and then methane gas from a cylinder (99%; Dong-A Gases, Korea) was injected into the bottles to a final concentration of 50,000 ppm [13]. In addition, DMS solution (99%; Acros Organics, Belgium) was injected into the bottles to a final concentration of 5,000 ppm. The serum bottles were incubated at 30°C, 40°C, and 50°C and 180 rpm in a shaking incubator. In order to prevent the stopper from falling out due to the expansion of the gas inside the serum bottle at 40°C and 50°C, 60 ml of the gas inside the serum bottle was removed using a syringe before putting the serum bottle into the incubator. The gas in the headspace of each serum bottle was periodically sampled using gas-tight syringes to measure the concentration of methane and DMS with a gas chromatography system (GC 7890, Agilent Technologies, USA) equipped with a 30 m × 320 μm × 1.8 μm capillary column (J&W Scientific, Inc., ISA) and a flame ionization detector (Agilent Technologies). The operating temperature of the oven, injector, and detector was 100°C, 230°C, and 230°C, respectively. The degradation rates for the methane and DMS were calculated as the reduction in the methane and DMS from their initial concentration to a concentration below 2,000 ppm and 500 ppm, respectively, divided by the incubation time per unit dry weight of the sample.

### Isolation and Identification of Methane- and DMS-Degrading Bacteria under Moderately Thermophilic Conditions

To isolate methane- and DMS-degrading bacteria at 40°C and 50°C, enrichment cultures were developed. Packing material samples taken at 0–15 cm, 15–30 cm, and 30–50 cm from the surface of the biocover on day 252 were mixed in equal amounts with each other. Five grams of the mixed sample was placed in each of two 600-ml serum bottles containing 20 ml of NMS medium. After sealing the bottles with butyl rubber stoppers, methane gas and DMS solution were injected to final concentrations of 50,000 ppm and 5,000 ppm, respectively. One bottle was incubated at 40°C, and the other was incubated at 50°C in a shaking incubator (180 rpm). When the concentrations of methane and DMS in the headspace of each bottle decreased below the detection limit, methane gas and DMS solution were re-injected and the bottle was re-incubated at 40°C or 50°C. After repeating this process five times, 10 ml of the 1^st^ enriched culture was transferred into 10 ml of fresh NMS medium in a 600 ml-serum bottle, and methane and DMS were injected into the bottle. Each bottle was incubated at 40°C or 50°C, and methane and DMS were re-supplied when their concentration fell below the detection limit before re-incubation. After repeating this process four times, 10 ml of the 2^nd^ enriched culture was transferred into 10 ml of fresh NMS medium, methane and DMS were supplied, and the bottle was incubated at 40°C or 50°C.

After repeating this replenishment four times, the 3^rd^ enriched culture was diluted with NMS medium, and the diluted culture was spread on NMS-agar (20 g/l) plates. The inoculated plates were incubated at 40°C or 50°C in a 5-L reactor, which was connected to a 3-L Tedlar bag containing 50,000 ppm of methane and 5,000 ppm of DMS. After incubation for 1–2 months, distinguishable colonies on the plates were carefully transferred to fresh NMS-agar plates and incubated in the same manner as described above. This process was repeated several times to produce four pure strains (HJ1, HJ2, HJ3, and HJ4) from the 40°C-enriched cultures and two pure strains (HJ5 and HJ6) from the 50°C-enriched cultures.

The simultaneous degradation of methane and DMS by the isolates in the serum bottles was evaluated at 40°C or 50°C using the same method as described in Section 2.6. To identify the isolates, genomic DNA samples were extracted using NucleoSpin Soil Kits (Macherey-Nagel GmbH Düren, Germany), and amplified with PCR using the primer set 340F (5’-TCCTACGGGAGGCAGCAG-3’) and 805R (5’-GACTACHVGGGTATCTAATCC-3’)[26]. The sequence data were deposited in the NCBI Sequence Read Archive (https://www.ncbi.nlm.nih.gov/) under the accession number MK577721 for the strain HY1, MK577720 for HY2, MK577719 for HY3, MK577718 for HY4, MK639430 for HY5, and MK577717 for HY6.

## Results and Discussion

### Variation in the Environmental Parameters

**Fig. 1:**
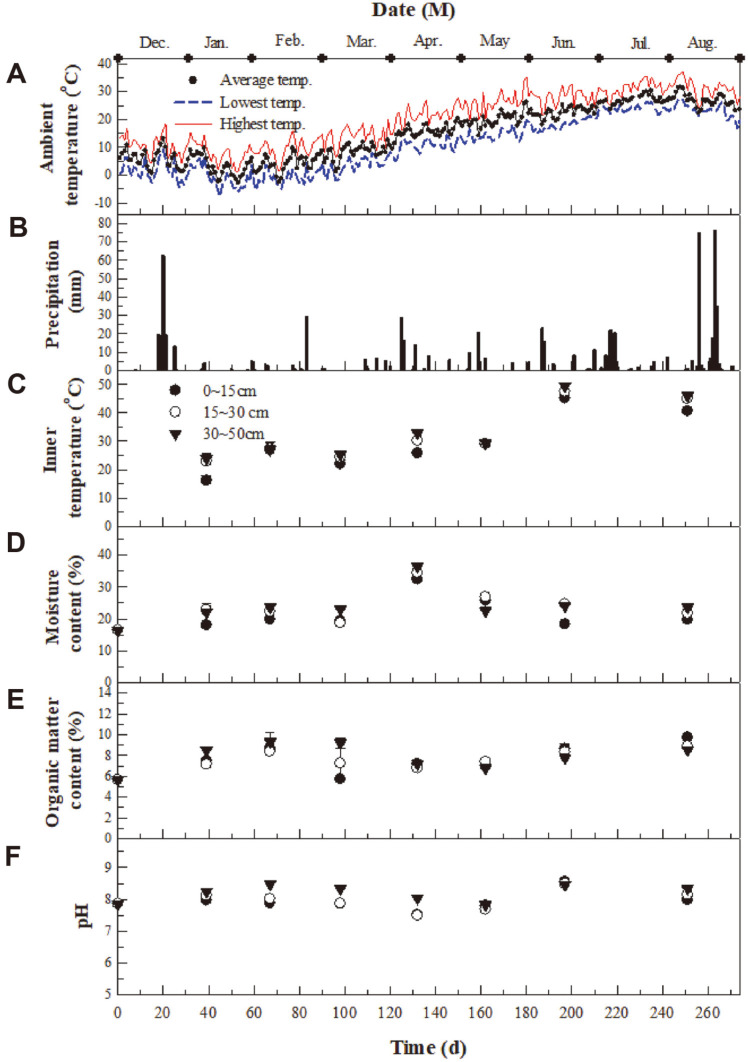
Time profiles of the ambient temperature (A), precipitation (B), internal temperature (C), moisture content (D), organic matter content (E), and pH (F) of the packing material in the biocover.

Environmental factors such as temperature, precipitation, moisture content, organic matter content, and pH affect biocover performance [10, 11, 27-32]. Fig. 1 presents the ambient temperature, precipitation, internal temperature, and physical characteristics of the packing material at different depths in the biocover. The average ambient temperature during winter (from December to February), spring (from March to May), and summer (from June to August) was –2.9–13.1ºC, 1.1–26.3ºC, and 18.0–31.5ºC, respectively (Fig 1A). Monthly precipitation was the highest in August (Fig. 1B). During winter and spring, the internal temperature tended to increase with greater distance from the surface of the biocover, with a range of 20–30ºC at midday (Fig. 1C), while that during summer was 41–49 ºC, even though the maximum ambient temperature was 21.4–37.0 ºC (Figs. 1A and 1C). There were four main reasons for this higher internal temperature: (1) the heat generated by biodegradation in the waste layer at the bottom of the biocover, (2) heat from the biodegradation of methane and odor compounds in the biocover, (3) heat from intense sunlight during the middle of the day, and (4) thermal insulation due to the polystyrene foam boards within the biocover (Fig. S1). Jung *et al*. [11] also reported that the temperature inside a biowindow was higher than the ambient temperature during winter and spring. The optimal temperature range for the growth and activity of methane- and odor-degrading bacteria has been shown to be 25–35°C [10, 11, 33, 34] because most of these species are mesophiles. Thus, the temperature inside the biocover during winter and spring was maintained within a suitable range for the simultaneous biodegradation of methane and odor compounds, but the internal temperature during the summer period was not favorable for mesophilic bacteria (Fig. 1C).

The moisture content, organic matter content, and pH of the packing material in the biocover are presented in Figs. 1D-1F. The moisture content in the biocover mostly remained at around 20–25% at all sample depths, though it did increase to 30% on day 124 due to heavy precipitation (Fig. 1D). Cho and Ryu [3] reported that the optimal moisture content for biocover performance was 25–50%. The moisture content of the biocover in the present study thus fell within a suitable range for the simultaneous degradation of methane and odor compounds. During summer, despite the high evaporation rate, the moisture content in the biocover was maintained at a suitable level due to the frequent precipitation (Figs. 1A, 1B, and 1D). The successful removal of methane and odor compounds within a biocover with a low organic matter content of 5–10% has been reported [10, 11, 18]. In addition, most methane- and/or odor-degrading bacteria exhibit optimal activity at a neutral pH [3, 34, 35]. Considering these previous results, the moisture content and pH of the biocover were favorable for methane- and odor-degrading bacteria.

### Methane and Odor Removal during Summer

**Fig. 2:**
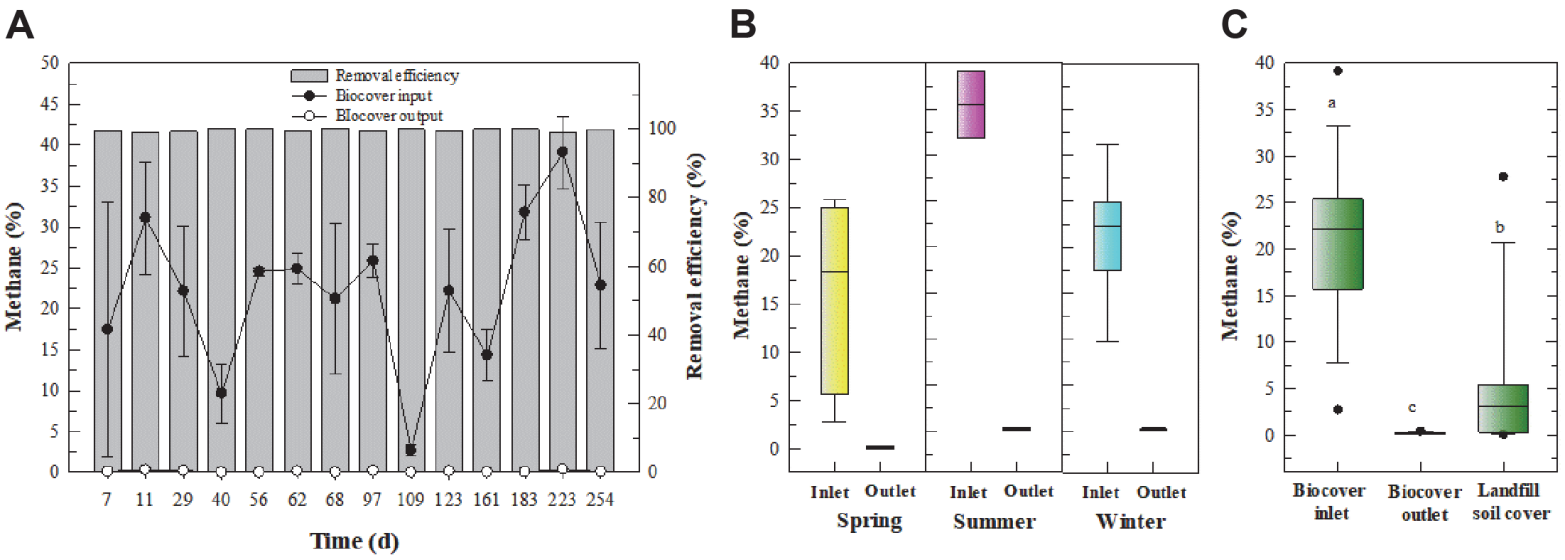
Removal of methane within the biocover. (**A**) Time profile for the methane concentration and removal efficiency. (**B**) Seasonal changes in the methane concentration at the biocover inlet and outlet. (**C**) Comparison of the methane concentration at the biocover inlet, the biocover outlet, and the landfill soil cover. The landfill soil cover was monitored for 240 days (Dec. 2016 to Aug. 2017) at the soil surface at the same landfill (Yun *et al*., 2017). In the box plot, the boxes represent the 25th, 50th (median), and 75th percentiles, and the error bars indicate the 10th and 90th percentiles. Different letters indicate a significant difference within each plot (*p* < 0.05).

Fig. 2A presents a time profile of the methane concentration and removal efficiency. The methane concentration at the biocover inlet was below 32% during winter and spring but ranged from 20–38% during summer. It is believed that the inlet methane concentration was higher during summer because the biodegradation of landfill waste was higher under thermophilic conditions. During the 254-day experimental period, the methane removal efficiency was over 98% despite the fluctuation in the inlet concentration (Fig. 2A). The average inlet methane concentration during winter, spring, and summer was 22.0%, 16.3%, and 31.3%, respectively, and the outlet concentration was 0.1%, 0.1%, and 0.2%, respectively (Fig. 2B). The methane removal by the biocover was compared with that of the soil cover at a site adjacent to the biocover [19]. The methane concentration at the surface of the biocover (*i.e.*, the outlet) remained below 0.4%, while that at the surface of the landfill soil cover fluctuated between 0.05% and 27.8% (Fig. 2B).

**Fig. 3:**
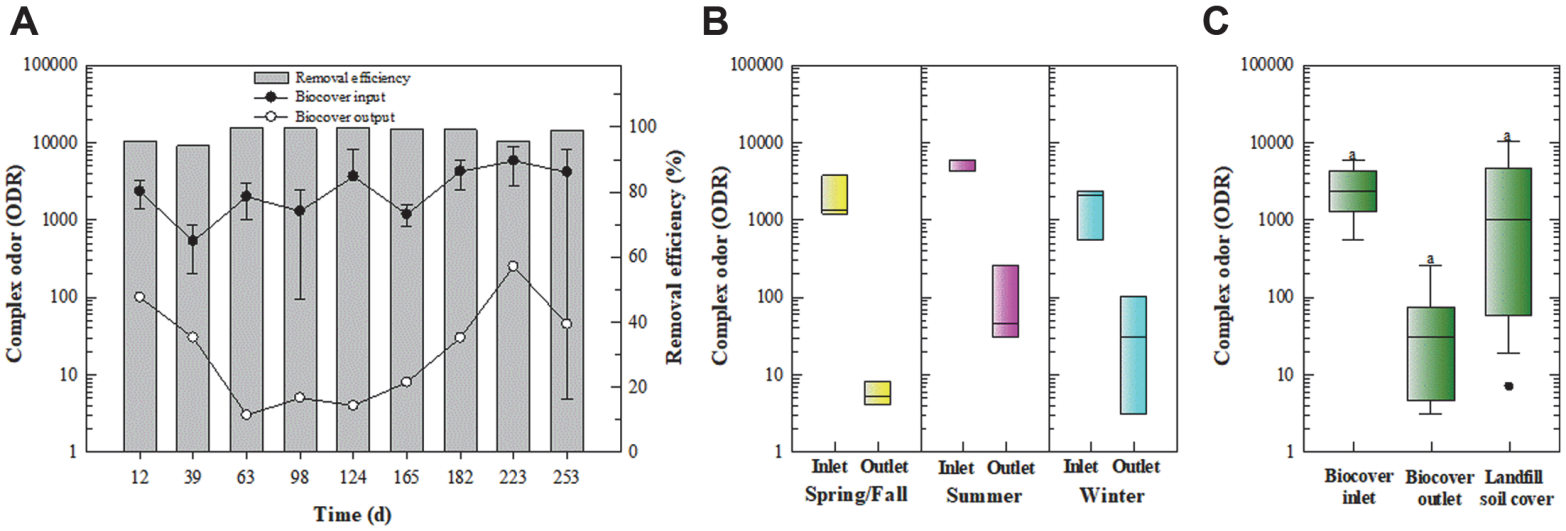
Removal of odor within the biocover. (**A**) Time profile for complex odor compounds (ODR) and the removal efficiency. (**B**) Seasonal changes in the ODR at the biocover inlet and outlet. (**C**) Comparison of the ODR at the biocover inlet, the biocover outlet, and the landfill soil cover. The landfill soil cover was monitored for 240 days (Dec. 2016 to Aug. 2017) at the soil surface at the same landfill (Yun *et al*., 2017). In the box plot, the boxes represent the 25th, 50th (median), and 75th percentiles, and the error bars indicate the 10th and 90th percentiles. Different letters indicate a significant difference within each plot (*p* < 0.05).

The concentration and removal efficiency for 22 odor compounds in the biocover are summarized in Table S1. Previous studies have reported that the key compounds that contribute to the complex odor intensity at the Gwangyang landfill are sulfur-containing compounds [10, 11, 18, 19, 36]. Similar to previous studies, sulfur-containing compounds were the primary contributors to the complex odor intensity in the present study (Table S1). The odor removal performance of the biocover is presented in Fig. 3. The average inlet ODR during summer (4735) was slightly higher than that during winter (1622) and spring (2046) (Figs. 3A and 3B). During summer, the average outlet ODR ranged from 30 to 100, with an average removal efficiency of 98%. The average odor removal efficiency was 96.6% during winter and 99.6% during spring. The ODR was 3–250 at the surface of the biocover across the entire sampling period but ranged from 7 to 10,000 at the surface of the landfill soil with no biocover (Fig. 3C).

In the pilot-scale biocover containing a mixture of soil, perlite, earthworm cast, and compost at a ratio of 6:2:1:1 (v/v), the methane and odor removal efficiency from the landfill gas was 85–96% and 93–98% during the spring and summer seasons, respectively [10]. A previous study reported that, across all seasons, odor removal by biocovers installed at a sanitary landfill ranged from 81 to 98% [18], which is similar to the biocover performance in the present study. In addition, there was no observed deterioration in methane or odor removal performance when the internal temperature of the biocover increased to more than 40ºC at midday during the summer season.

### Bacterial Community Structure during Summer

**Fig. 4:**
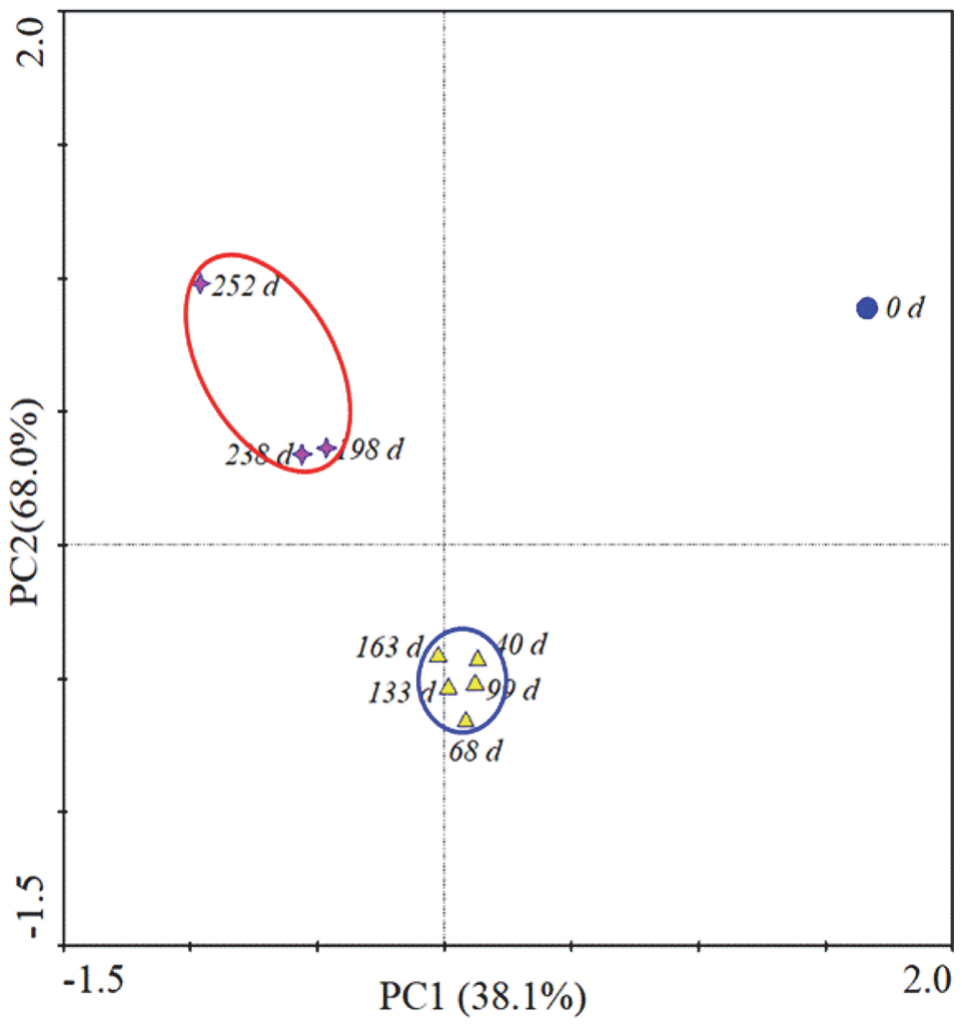
Principal component analysis (PCA) of the structure of the bacterial community in the biocover. The community structure was analyzed in duplicate.

Table 1 summarizes the bacterial community in the biocover. The number of OTUs increased from 545 to 1060 between day 0 and day 113 and then settled down to 441–686. The Shannon index, which is used to evaluate the diversity of a bacterial community, fluctuated between 4.703 and 5.587. The Shannon index during summer was 4.703–5.470, while that during winter and spring was 4.839–5.587, showing that the diversity of the bacterial community did not decrease significantly during summer. Principal component analysis (PCA) was also conducted to compare the structure of the bacterial community by season (Fig. 4), leading to the creation of three clear groups. The composition of the community during summer (days 198–252) differed from that during the winter and spring seasons (days 40–163).

Table 2 presents the relative abundance of the bacterial genera in the biocover at each sampling point. The correlation between the bacterial community and environmental parameters is shown in Table S2. The generation of a thermophilic environment in the biocover during summer promoted the growth of thermophilic heterotrophs, including *Bellilinea*, *Rhodothermus*, *Thermanaerothrix*, and *Thermomarinilinea* (Tables 2 and S2)[37-40]. Many *Rhodothermus* spp. experience large fluctuations in environmental conditions, and these can utilize a wide variety of carbon sources using thermostable enzymes (Bjornsdottir *et al*., 2006), suggesting that the genus may contribute to odor degradation during summer. In contrast, because *Bellilinea*, *Thermanaerothrix*, and *Thermomarinilinea* are obligate anaerobes that can also produce methane via anaerobic metabolism, it is believed that they contribute little to methane or odor degradation [37, 39, 40]. In addition, even though the genus *Ornatilinea* is known as strictly anaerobic, mesophilic, and organotrophic, its dominance increased during summer because some of its species exhibit optimal growth at 42–45°C [41].

The relative abundance of the genus *Hyphomicrobium* increased from 0.35–1.10% during winter and spring to 0.94–6.42% during summer (Tables 2 and S2). *Hyphomicrobium*, a methylotrophic genus, can degrade sulfur-containing odor compounds such as DMS and hydrogen sulfide as well as methane and methanol [42-44]. This genus has previously been identified as a dominant genus in landfill biocovers for methane and odor mitigation [11, 18]. *Hyphomicrobium* has also been reported to be the dominant genus in thermophilic processes, including sludge composting and thermophilic biotrickling filtering, and is a mesophilic aerobe [45, 46]. Thus, this genus is likely to have played a major role in the degradation of methane and odor in the biocover even during summer due to its thermotolerant characteristics.

The pattern for the genus *Ohtaekwangia* during the experimental period was similar to that of *Hyphomicrobium*. The dominance of *Ohtaekwangia* increased with higher ambient temperatures, with a range of 0.90–1.70% during winter, 1.70–4.10% during spring, and 1.50–7.28% during summer (Tables 2 and S2). Similar to our results, this genus has been detected during the thermophilic, maturation, and mesophilic stages during olive mill waste composting, even though *Ohtaekwangia* is an aerobic mesophile [47]. *Ohtaekwangia* has been reported to co-exist with methanotrophs such as *Methylocystis* and *Methylosinus* in a biofilter for methane mitigation [48]. In addition, *Ohtaekwangia* was one of the dominant bacterial genera in a biocomplex textile installed at a sanitary landfill [36]. Based on these results, *Ohtaekwangia* may be directly or indirectly associated with methane and odor biodegradation across all seasons.

The relative abundance of *Bacillus* fluctuated widely, with a range of 1.00–8.53% across all seasons (Tables 2 and S2). Mesophilic and thermophilic *Bacillus* species have been reported [49, 50], while thermotolerant methylotrophic *Bacillus* strains that can utilize the RuMP pathway for formaldehyde fixation have been isolated [51, 52]. This genus has been identified as one of the most dominant in biocovers for odor control [18]. These results suggest that the genus *Bacillus* is an important player in methane and odor mitigation during both the summer and non-summer seasons.

The most dominant methanotrophs were *Methylocaldum* during summer and *Methylobacter* during winter and spring (Table 2). The relative abundance of *Methylobacter* increased to 37.30–41.90% in winter and spring but fell to 6.60% during summer, while that of *Methylocaldum* tended to increase with higher ambient temperatures, reaching 24.86% and 23.40% on days 238 and 252, respectively (Tables 2 and S2). Thermophilic *Methylocaldum* has previously been isolated from a high-temperature hot spring and is known to have a broad range of growth temperatures between 30°C to 61°C, with optimal growth at 55°C [53]. In addition, thermotolerant and thermophilic *Methylocaldum* species, with an optimal growth temperature above 40°C, have been isolated from agricultural soil and the effluent of a hot spring [54]. *Methylobacter* and *Methylocaldum* were also the dominant methanotrophs in a pilot-scale biocover, biowindow, and biocomplex textile installed at a landfill [10, 11, 36]. These results suggest that *Methylocaldum*, which adapts well to high temperatures, plays a key role in methane oxidation during the summer season, while *Methylobacter* is important during winter and spring (Tables 2 and S2).

### Simultaneous Removal of Methane and DMS by the Packing Material under Moderately Thermophilic Conditions

**Fig. 5:**
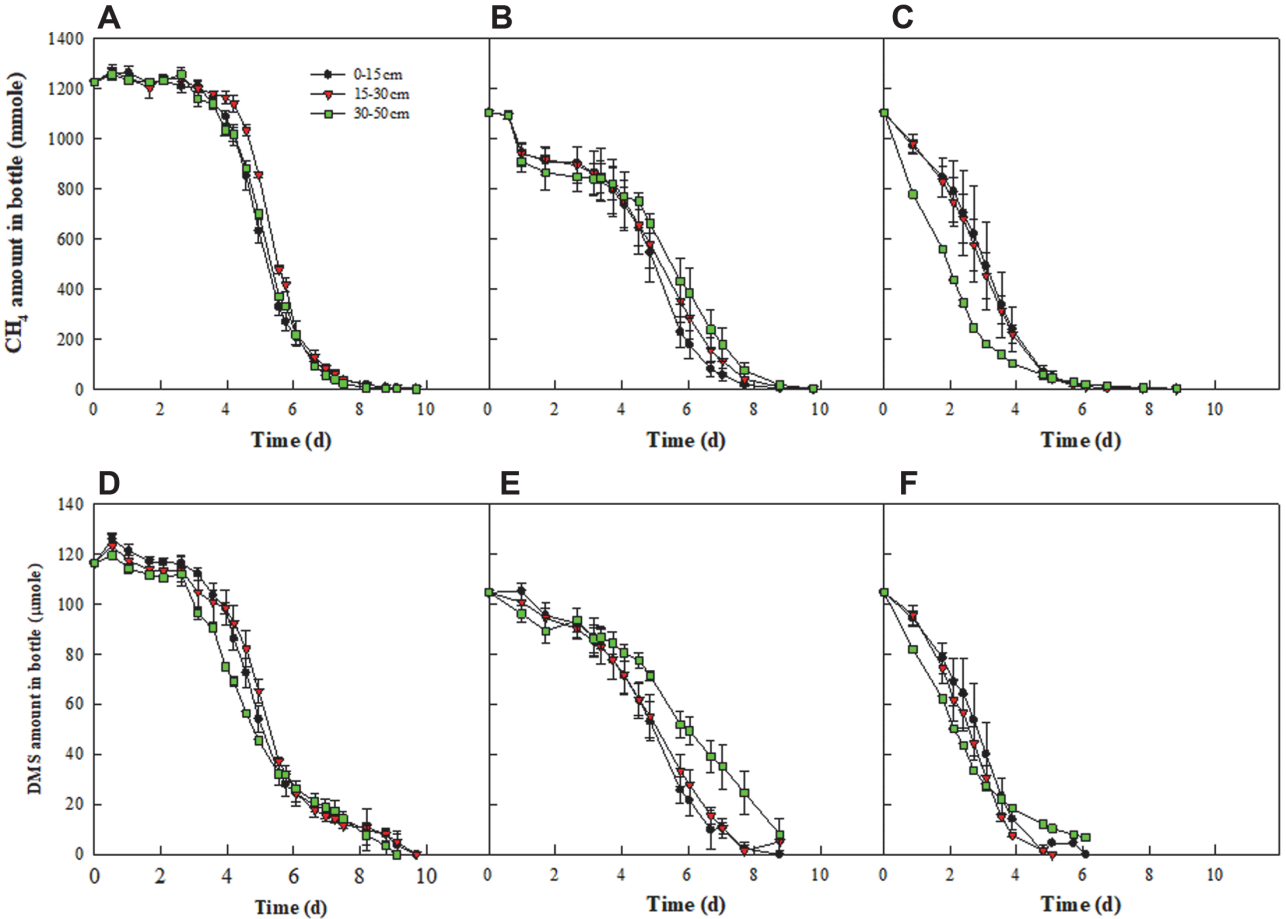
Time profiles for methane (A–C) and DMS (D–F) degradation by the packing material sampled at 0– 15, 15–30, and 30–50 cm from the surface of the biocover. Incubation temperatures: (**A**) and (**D**) 30°C, (**B**) and (**D**) 40°C, and (**C**) and (**F**) 50°C.

Fig. 5 presents time profiles for methane and DMS degradation by the packing material sampled during summer at 30°C, 40°C, and 50°C. All of the packing material samples from the top (0–15 cm), middle (15–30 cm), and bottom (30–50 cm) layers of the biocover simultaneously degraded methane and DMS under moderately thermophilic conditions (40–50°C) and mesophilic conditions (30°C). Table 3 presents the degradation rates for methane and DMS at different temperatures. The degradation rate for both methane and DMS at 50°C was significantly higher than at 30°C and 40°C and increased with a greater sampling depth, with the degradation rate for methane and DMS highest at 30–50 cm (3.059 ± 0.074 and 0.256 ± 0.005 μmol·g-dry sample^-1^·h^-1^, respectively). Because the internal temperature during summer increased to 41–49ºC at midday during summer (Fig. 1C), the methane and DMS degradation was higher under moderately thermophilic conditions than under mesophilic conditions. Degradation was also assumed to be highest at 30–50 cm because the internal temperatures tended to rise as the depth of the biocover increased (Fig. 1C).

### Isolation and Identification of Thermophilic Methanotrophs and Simultaneous Removal of Methane and DMS

**Fig. 6:**
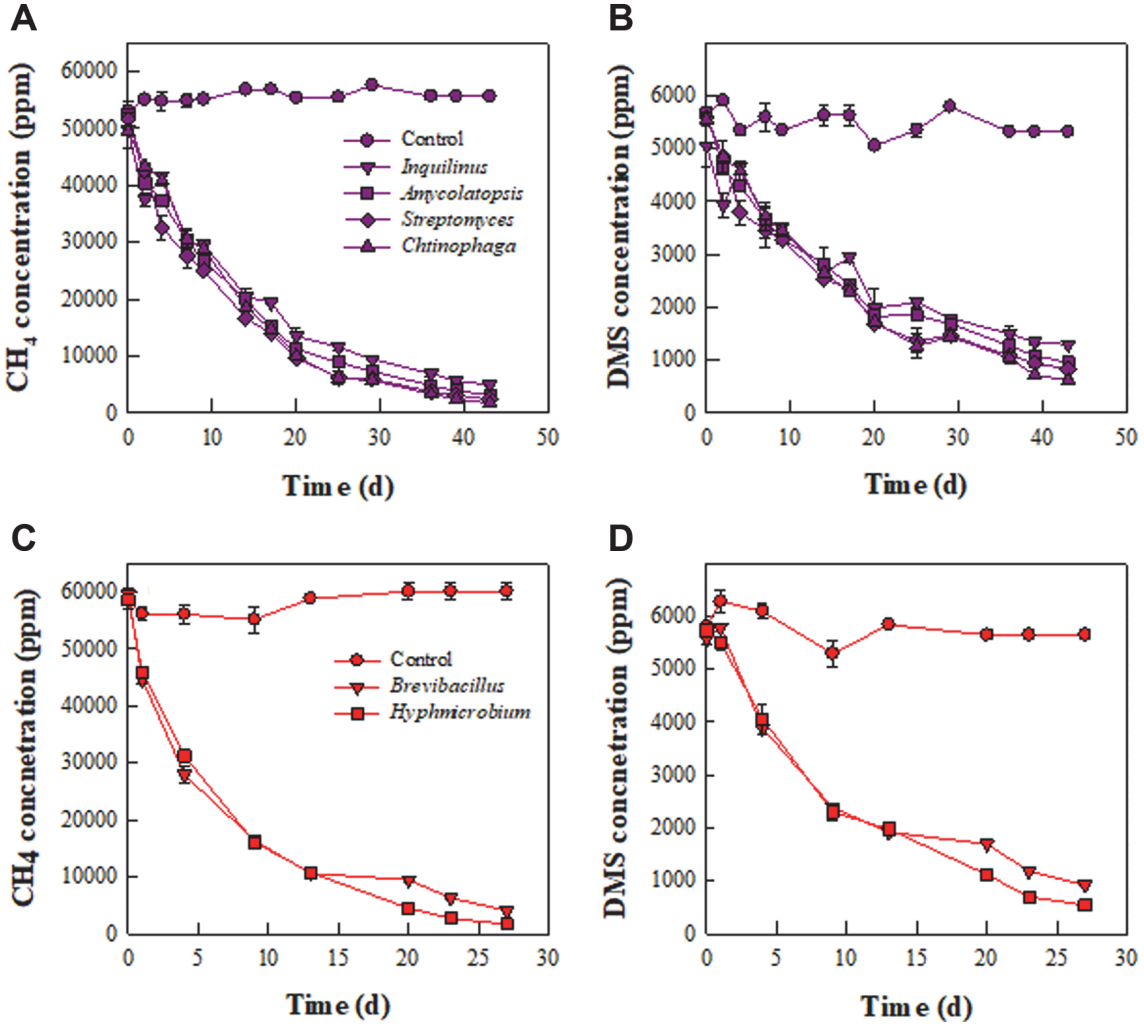
Time profiles for methane (A and C) and DMS (B and D) degradation by the isolates from the biocover. Incubation temperatures: (**A**) and (**B**) 40°C and (**C**) and (**D**) at 50°C.

Four methane- and DMS-degrading bacterial species – *Inquilinus* sp. HJ1, *Amycolatopsis* sp. HJ2, *Streptomyces* sp. HJ3, and *Chitinophaga* sp. HJ4 – were isolated at 40°C (Figs. 6A and 6B), though they were not able to degrade methane or DMS at 50°C (data not shown). *Brevibacillus* sp. HJ5 and *Hyphomicrobium* sp. HJ6, which were isolated from the enrichment culture at 50°C, simultaneously degraded methane and DMS at 50°C and at 40°C (Figs. 6C and 6D). Methane and DMS degradation by the isolates at 50°C was faster than at 40°C. This is consistent with the experimental results, which found that degradation by the packing material was greater at 50°C than at 40°C (Table 3).

The bacterial community results reveal that the abundance of *Hyphomicrobium* during summer was higher during the non-summer seasons (Table 2). This genus can utilize C1 compounds such as methane and methanol and deodorize H_2_S, methanethiol, and DMS [42-44] and has previously been reported to be dominant in biological treatment systems for the removal of methane and DMS [11, 36]. The abundance of *Brevibacillus* in the biocover ranged from 0.01% to 0.04% (data not shown). Some *Brevibacillus* species are thermophilic and others are mesophilic [55, 56], while Chebbi *et al*. [56] reported that *Brevibacillus* sp. CAT37 degraded malodorous thiols. The abundance of *Streptomyces*, a representative Actinobacteria genus, in the biocover was 0.52–1.40%during the non-summer seasons and 0.57–0.91% during summer (Table 2). A methane-utilizing *Streptomyces* species has been isolated from paddy soil [57], and *Streptomyces griseus* has been used in a biofilter for odor removal [58]. Some *Streptomyces* strains also exhibit thermotolerance [59].

Although three strains belonging to *Inquilinus*, *Chitinophaga*, and *Amycolatopsis* were isolated from the enrichment cultures in this study (Fig. 6), their abundance in the bacterial community was below 0.01% (data not shown). The *Inquilinus* sp. isolated from a ginseng soil field is a strictly aerobic, mesophilic heterotroph [60], while another *Inquilinus* strain has been identified as a plant-growth-promoting endophytic bacterium [61]. To the best of our knowledge, no previous studies have reported its thermotolerance or its methane or odor degradation ability. A *Chitinophaga* strain isolated from soil was shown to be an aerobic heterotroph that could grow at 15–42°C [62]. *Chitinophaga* has also been detected as one of the dominant genera in a sludge-composting process [45]. *Amycolatopsis methanolica* is a facultatively methylotrophic actinomycete [63], while *Amycolatopsis rugosa* can grow at 10–45°C and decarboxylate odorous acids such as acetate and propionate [64].

Except for *Inquilinus*, a review of past research confirmed that the genera of the isolated strains exhibit heat resistance and/or thermophilic properties. Previous studies indicate that *Hyphomicrobium*, *Streptomyces*, and *Amycolatopsis* have methylotrophic characteristics, meaning that they utilize methane as a carbon source [42-44, 57, 63]. Previous research has also found that *Hyphomicrobium* and *Brevibacillus* have strong potential for DMS degradation [42-44, 56]. In the present study, only preliminary results for methane and DMS degradation at 40°C and 50°C by the isolated strains were presented. Further investigation is required to determine the mechanisms underlying methane and DMS degradation by the isolates under moderately thermophilic conditions.

It took around 8 to 10 days for the packing material to completely degrade the methane and DMS at different depths in the biocover (Fig. 5). However, it took more than 40 days for methane and DMS to be completely decomposed by pure bacteria isolated from the enrichment culture at 40°C (Figs. 6A and 6B). In addition, the degradation rates for *Brevibacillus* sp. HJ5 and *Hyphomicrobium* sp. HJ6, which are capable of decomposing methane and DMS at 50°C, were also slower than for the packing material (Fig. 6). This suggests that a variety of bacteria are involved in the degradation of methane and DMS in the packing material, leading to a higher degradation rate. In general, the higher the diversity of a microbial community, the more diverse its functions and the higher its functional stability against disturbance [65, 66]. In this study, the bacterial diversity in the biocover was maintained within a certain range (Table 1). The bacterial community results show that the dominant bacterial species involved in the degradation of methane and odor changed as the environmental conditions changed. For example, as the ambient temperature increased, the major methane-oxidizing bacteria were *Methylocaldum* spp., replacing *Methylobacter* spp. Due to the change in the dominant species and the maintenance of bacterial diversity, the methane and odor removal performance of the biocover was not significantly disrupted by changes in the environmental conditions, such as the ambient temperature and precipitation.

In this study, the simultaneous removal of methane and odor in the biocover was compared between the summer and non-summer seasons. The biocover performance for the methane and odor compounds did not deteriorate even when the internal temperature of the biocover increased to more than 40°C at midday during summer. The packing material sampled from the biocover in summer was able to degrade methane and DMS at 40–50°C, while the isolated bacteria simultaneously degraded methane and DMS at 40°C and/or 50°C. The diversity of the bacterial community in the biocover also remained constant regardless of the season, though the relative abundance of thermotolerant and thermophilic bacteria increased during summer. The major methane-oxidizing bacterial group shifted from *Methylobacter* during the non-summer seasons to *Methylocaldum* during summer. These results indicate that the methane and odor removal ability of a biocover can be maintained under moderately thermophilic conditions due to the replacement of the main species and the maintenance of bacterial diversity. Taken as a whole, the information regarding the bacterial community dynamics under moderately thermophilic conditions revealed in the present study could be used to design biological treatment systems for methane and odor mitigation during summer and/or in subtropical countries.

## Supplemental Materials

Supplementary data for this paper are available online only at http://jmb.or.kr.



## Figures and Tables

**Table 1:** Bacterial community analysis results for the biocover.

Sampling time (d)	No. of OTUs ^a^	Chao1^b^	Shannon^c^	Good coverage^d^
0	545	648	5.445	0.998
40	708	873	5.227	0.996
68	874	983	4.839	0.999
99	996	1160	5.411	0.997
133	1060	1193	5.587	0.998
163	650	801	4.890	0.989
**198**	**441**	**603**	**5.373**	**0.985**
**238**	**686**	**818**	**4.703**	**0.998**
**252**	**673**	**885**	**5.470**	**0.989**

^a^Operational taxonomic units

^b^Chao1 is used to evaluate bacterial community richness.

^c^The Shannon index is used to evaluate the diversity within a bacterial community.

^d^Good coverage is calculated as C=1-(s/n), where s is the number of unique OTUs and n is the number of individuals in the sample. This index provides a relative measure of how well the sample represents the wider community.

**Table 2:** Comparison of the relative abundance of bacterial genera in the biocover.

Genus		Sampling time (d)								

		0	40	68	99	133	163	**198**	**238**	**252**
Non-Methanotroph	*Acinetobacter*	15.70	-^*^	-	-	-	1.10	**-**	**-**	**-**
	*Actinomadura*	-	0.90	-	-	-	-	**-**	**1.13**	**-**
	*Actinophytocola*	-	-	-	-	-	-	**-**	**1.16**	**-**
	*Advenella*	1.20	3.00	1.90	0.90	-	-	**-**	**-**	**-**
	*Arthrobacter*	8.00	-	-	1.20	-	-	**-**	**-**	**-**
	*Bacillus*	1.00	-	-	-	-	2.20	**1.10**	**8.53**	**1.00**
	*Bellilinea*	-	-	-	-	-	-	**4.00**	**-**	**1.90**
	*Brevibacterium*	-	-	-	-	-	-	**-**	**1.88**	**-**
	*Cellulosimicrobium*	-	-	-	1.50	-	-	**-**	**-**	**-**
	*Chryseolinea*	-	-	-	-	-	1.10	**-**	**-**	**-**
	*Desertibacter*	-	-	-	-	-	-	**-**	**-**	**1.70**
	*Dokdonella*	-	-	-	-	1.60	-	**-**	**-**	**-**
	*Homoserinibacter*	1.10	-	1.20	1.30	1.20	-	**-**	**-**	**-**
	*Hydrogenophaga*	-	-	1.30	-	-	-	**-**	**-**	**-**
	*Hyphomicrobium*	0.44	0.46	0.35	0.49	0.52	1.10	**1.20**	**6.42**	**0.94**
	*Ignavibacterium*	-	-	-	-	-	-	**-**	**-**	**1.80**
	*Lascolabacillus*	-	-	1.20	-	-	-	**-**	**-**	**-**
	*Luteimonas*	-	7.50	1.60	1.70	1.20	2.00	**-**	**4.80**	**-**
	*Microbispora*	1.50	-	-	-	-	-	**-**	**-**	**-**
	*Nonomuraea*	-	4.90	2.00	1.80	1.20	0.90	**-**	**-**	**-**
	*Ohtaekwangia*	-	1.70	0.90	1.70	1.70	4.10	**1.50**	**7.28**	**5.90**
	*Ornatilinea*	-	-	-	-	-	-	**2.20**	**-**	**2.70**
	*Pedobacter*	8.10	-	-	-	-	-	**-**	**-**	**-**
	*Planococcus*	8.30	-	-	1.20	-	-	**-**	**-**	**-**
	*Porticoccus*	2.90	-	-	-	-	-	**-**	**-**	**-**
	*Pseudomonas*	3.80	1.10	-	-	-	-	**-**	**-**	**-**
	*Pseudoxanthomonas*	-	1.00	-	-	-	-	**-**	**-**	**-**
	*Rhodothermus*	-	-	-	-	-	-	**4.60**	**-**	**6.10**
	*Rummeliibacillus*	1.20	-	-	-	-	1.70	**-**	**-**	**-**
	*Serpens*	15.70	27.20	13.90	13.40	12.40	7.30	**18.10**	**-**	**2.00**
	*Streptomyces*	0.70	1.70	0.90	1.40	0.52	0.64	**0.28**	**0.91**	**0.57**
	*Thermanaerothrix*	0.80	1.40	-	1.00	1.50	-	**4.80**	**0.83**	**12.30**
	*Thermomarinilinea*	-	-	-	-	-	-	**13.90**	**3.55**	**17.90**
Methanotroph	*Methylocaldum*	1.30	2.40	0.90	1.60	2.40	5.80	**9.00**	**24.86**	**23.40**
	*Methylococcus*	-	-	-	0.40	0.80	0.80	**0.20**	**3.46**	**-**
	*Methylobacter*	0.80	15.90	41.90	37.80	37.30	37.70	**13.00**	**6.60**	**6.60**
	*Methylomicrobium*	-	0.10	-	0.40	0.10	0.10	**-**	**-**	**-**
	*Methylosarcina*	-	-	0.10	0.20	0.50	0.60	**-**	**-**	**-**
	*Methylomonas*	-	-	-	-	0.20	-	**-**	**-**	**-**
	*Methylocystis*	-	-	-	0.10	0.20	0.10	**0.10**	**-**	**-**
Others		27.46	30.73	31.85	31.91	36.66	32.76	**26.02**	**28.59**	**15.19**
Total		100	100	100	100	100	100	**100**	**100**	**100**

*-, Less than 0.1%

**Table 3:** Comparison of the methane and DMS degradation rate by the packing material at 30, 40, and 50°C.

Temp. (℃)	Sampling layer (cm)	Degradation rate for the packing material (μmol·g-dry sample^-1^·h^-1^)	

		CH_4_	DMS
30℃	0–15	1.502 ± 0.060 ^C, D^*	0.136 ± 0.001 ^D, E^
	15–30	1.614 ± 0.060 ^C^	0.148 ± 0.004 ^D^
	30–50	1.682 ± 0.074 ^C^	0.154 ± 0.005 ^D^
40℃	0–15	1.388 ± 0.060 ^C, D^	0.124 ± 0.004 ^E, F^
	15–30	1.335 ± 0.060 ^D^	0.125 ± 0.004 ^E, F^
	30–50	1.407 ± 0.060 ^C, D^	0.110 ± 0.004 ^F^
50℃	0–15	2.052 ± 0.060 ^B^	0.208 ± 0.004 ^C^
	15–30	2.101 ± 0.060 ^B^	0.228 ± 0.004 ^B^
	30–50	3.059 ± 0.074 ^A^	0.256 ± 0.005 ^A^

*Different letters denote a significant difference (*n* = 3, *p* < 0.05).
