# Supplementary material for: Characterization of the Bacterial Community Associated with Methane and Odor in a Pilot-Scale Landfill Biocover under Moderately Thermophilic Conditions
Source: J Microbiol Biotechnol. 2021 Apr 21;31(6):803–14. doi: 10.4014/jmb.2103.03005 (PMC9705922; doi:10.4014/jmb.2103.03005)
Supplement: Supplementary file 1 [file jmb-31-6-803-supple.pdf]

J. Microbiol. Biotechnol.
https://doi.org/10.4014/jmb.2103.03005

[Supplementary materials]

Characterization of the bacterial community associated with methane and odor in a pilot-scale landfill biocover under moderately thermophilic conditions

Hyoju Yang[1], Hyekyeng Jung[1], Kyungcheol Oh[2], Jun-Min Jeon[2], Kyung-Suk Cho[**]

[a]Department of Environmental Science and Engineering, Ewha Womans University, Seoul 03760, Republic of Korea

[b]Green Environmental Complex Center, Suncheon 57992, Republic of Korea

* Correspondence to:
Phone: +82-2-32772393
E-mail: kscho@ewha.ac.kr

Table S1. Concentrations and removal efficiencies for odor compounds.

	Time (d)	Ammonia	Trimethyl amine	Hydrogen sulfide	Methane-thiol	Dimethyl sulfide	Dimethyl disulfide	Acet aldehyde	Propion aldehyde	Butyl aldehyde	*i*-Valeric aldehyde	*n*-Valeric aldehyde	Methyl ethyl ketone	Methyl isobutyl ketone	Butyl acetate	*i*-Butyl alcohol	Styrene	Toluene	Xylene	*n*-Butyric acid	Propionic acid	*i*-Valeric acid	*n*-Valeric acid
**Biocover inlet conc. (ppb)**	12	365.9	bdl	7922.0	138.0	91.4	8.8	2.3	bdl	bdl	0.9	bdl	14.2	173.9	180.7	8.8	6.2	23.0	10.4	bdl	3.6	bdl	0.9
	39	180.2	bdl	13.1	15.8	53.6	25.6	2.0	bdl	0.4	bdl	bdl	bdl	184.6	308.3	bdl	bdl	bdl	bdl	bdl	0.7	bdl	bdl
	63	174.1	bdl	15,220.9	234.5	40.2	13.1	3.4	2.9	0.1	4.5	bdl	84.6	861.1	408.8	bdl	bdl	bdl	bdl	bdl	12.1	bdl	bdl
	98	180.8	0.2	1164.4	92.7	53.5	8.4	1.6	1.9	0.7	bdl	bdl	3.8	49.5	129.3	0.3	bdl	bdl	bdl	bdl	bdl	0.3	0.9
	124	261.8	0.1	1252.5	15.6	25.6	6.1	4.2	bdl	bdl	bdl	bdl	7.1	73.8	192.5	0.5	bdl	bdl	bdl	bdl	21.2	22.5	27.7
	165	406.7	6.9	1613.6	41.0	130.8	18.6	3.5	0.7	0.6	bdl	bdl	5.0	39.2	70.8	1.5	bdl	bdl	bdl	6.5	11.1	bdl	bdl
	**182**	**345.1**	**bdl**	**3591.9**	**217.9**	**28.0**	**11.8**	**3.5**	**bdl**	**bdl**	**bdl**	**bdl**	**14.5**	**518.2**	**738.0**	**1.9**	**5.6**	**bdl**	**bdl**	**bdl**	**0.0**	**bdl**	**bdl**
	**223**	**634.8**	**3.3**	**3317.8**	**195.2**	**28.0**	**1.2**	**6.0**	**0.7**	**0.8**	**0.5**	**bdl**	**16.3**	**272.2**	**560.8**	**4.5**	**4.9**	**bdl**	**bdl**	**bdl**	**1.2**	**0.9**	**1.1**
	**253**	**323.7**	**0.1**	**1962.7**	**46.6**	**12.1**	**0.0**	**39.7**	**2.6**	**7.4**	**2.0**	**bdl**	**588.0**	**320.0**	**1099.9**	**9.8**	**15.6**	**bdl**	**bdl**	**bdl**	**3.1**	**2.7**	**1.8**
**Biocover outlet (surface) conc. (ppb)**	12	964.2	1.7	2.8	2.2	1.9	1.2	4.1	0.8	0.3	bdl	bdl	bdl	4.8	12.4	5.5	2.2	7.7	4.4	bdl	bdl	bdl	bdl
	39	255.6	bdl	38.6	4.5	3.6	11.3	1.2	bdl	bdl	bdl	bdl	bdl	2.8	bdl	bdl	bdl	bdl	bdl	bdl	bdl	bdl	bdl
	63	197.7	bdl	1.7	1.5	0.3	0.4	2.3	2.5	6.2	5.5	bdl	20.4	998.9	68.0	bdl	bdl	bdl	bdl	bdl	bdl	bdl	bdl
	98	146.1	0.3	3.8	2.6	2.7	2.9	7.5	2.7	1.2	bdl	bdl	2.6	4.8	bdl	bdl	bdl	bdl	bdl	bdl	1.2	0.9	1.5
	124	647.3	0.2	1.2	1.0	1.0	0.4	5.8	bdl	bdl	bdl	bdl	4.1	13.5	bdl	bdl	0.6	bdl	bdl	bdl	11.8	bdl	21.5
	165	797.7	2.6	1.5	0.9	1.4	0.7	5.3	2.2	0.9	bdl	1.2	3.8	5.2	11.5	bdl	bdl	bdl	bdl	6.0	bdl	bdl	bdl
	**182**	**312.7**	**0.2**	**99.7**	**6.9**	**0.7**	**13.8**	**8.7**	**bdl**	**bdl**	**bdl**	**bdl**	**4.1**	**4.8**	**2.5**	**bdl**	**bdl**	**bdl**	**bdl**	**bdl**	**bdl**	**bdl**	**bdl**
	**223**	**577.5**	**0.7**	**499.2**	**3.2**	**bdl**	**0.5**	**7.9**	**bdl**	**3.1**	**0.8**	**bdl**	**5.2**	**10.7**	**21.8**	**6.2**	**bdl**	**bdl**	**bdl**	**bdl**	**1.0**	**0.9**	**1.1**
	**253**	**610.7**	**0.2**	**0.5**	**5.7**	**10.4**	**19.0**	**4.2**	**0.2**	**2.8**	**bdl**	**bdl**	**11.4**	**110.5**	**54.6**	**30.3**	**9.9**	**bdl**	**bdl**	**bdl**	**3.2**	**2.8**	**2.8**
**Removal efficiency (%)**	12	-	-	100.0	98.4	97.9	86.1	-	-	-	100.0	-	100.0	97.3	93.2	97.1	63.7	66.7	59.1	-	100.0	-	100.0
	39	-	-	-	71.4	93.4	55.9	36.9	-	100.0	-	-	-	98.5	100.0	-	-	-	-	-	100.0	-	-
	63	-	-	100.0	99.4	99.2	97.2	33.4	13.9	-	-	-	75.9	-	83.4	-	-	-	-	-	100.0	-	-
	98	19.2	-	99.7	97.2	94.9	65.7	-	-	-	-	-	29.9	90.4	100.0	100.0	-	-	-	-	-	-	-
	124	-	-	99.9	93.8	96.2	93.3	-	-	-	-	-	42.1	81.7	100.0	100.0	-	-	-	-	44.5	100.0	22.2
	165	-	63.3	99.9	97.9	99.0	96.1	-	-	-	-	-	24.3	86.8	83.8	100.0	-	-	-	8.0	100.0	-	-
	**182**	**9.4**	-	**97.2**	**96.8**	**97.5**	-	-	-	-	-	-	**71.8**	**99.1**	**99.7**	**100.0**	**100.0**	-	-	-	-	-	-
	**223**	**9**	**80.2**	**85.0**	**98.3**	**100.0**	**60.9**	-	**100.0**	-	-	-	**67.9**	**96.1**	**96.1**	-	**100.0**	-	-	-	-	-	-
	**253**	-	-	**100.0**	**87.7**	**14.4**	**0.0**	**89.3**	**91.9**	**62.4**	**100.0**	-	**98.1**	**65.5**	**95.0**	-	**36.5**	-	-	-	-	-	-

Bdl, Below detection limit

Table S2. Correlations between the bacterial community and environmental parameters.

Bacterial community	Genus	Highest ambient temp.	Lowest ambient temp.	Average ambient temp.	Biocover inner temp.	Biocover water content	Biocover organic content	Biocover pH
Non-Methanotroph	*Acinetobacter*	**-0.307**[a]	**-0.219**	**-0.219**	**-0.219**	**-0.495**	**-0.726**	**-0.098**
	Actinomadura	**0.012**	**0.107**	**0.107**	**0.107**	**-0.137**	**0.307**	**0.384**
	Actinophytocola	**0.408**	**0.449**	**0.449**	**0.449**	**-0.128**	**0.492**	**0.269**
	Advenella	**-0.871**	**-0.805**	**-0.805**	**-0.805**	**-0.374**	**-0.261**	**0.245**
	Arthrobacter	**-0.349**	**-0.275**	**-0.275**	**-0.275**	**-0.565**	**-0.739**	**-0.086**
	Bacillus	**0.539**	**0.583**	**0.583**	**0.583**	**-0.120**	**0.478**	**0.241**
	Bellilinea	**0.529**	**0.527**	**0.527**	**0.527**	**0.068**	**0.391**	**0.498**
	Brevibacterium	**0.408**	**0.449**	**0.449**	**0.449**	**-0.128**	**0.492**	**0.269**
	Cellulosimicrobium	**-0.189**	**-0.308**	**-0.308**	**-0.308**	**-0.342**	**-0.114**	**-0.064**
	Chryseolinea	**0.176**	**0.138**	**0.138**	**0.138**	**0.253**	**-0.065**	**-0.295**
	Desertibacter	**0.523**	**0.549**	**0.549**	**0.549**	**-0.037**	**0.256**	**-0.352**
	Dokdonella	**0.007**	**-0.037**	**-0.037**	**-0.037**	**0.806**	**-0.291**	**-0.531**
	Homoserinibacter	**-0.574**	**-0.652**	**-0.652**	**-0.652**	**-0.074**	**-0.483**	**-0.348**
	Hydrogenophaga	**-0.464**	**-0.542**	**-0.542**	**-0.542**	**-0.039**	**0.267**	**0.041**
	Hyphomicrobium	**0.529**	**0.566**	**0.566**	**0.566**	**-0.085**	**0.549**	**0.294**
	Ignavibacterium	**0.523**	**0.549**	**0.549**	**0.549**	**-0.037**	**0.256**	**-0.352**
	Lascolabacillus	**-0.421**	**-0.456**	**-0.456**	**-0.456**	**-0.087**	**0.306**	**0.119**
	Luteimonas	**-0.315**	**-0.248**	**-0.248**	**-0.248**	**-0.026**	**0.133**	**0.257**
	Microbispora	**-0.318**	**-0.227**	**-0.227**	**-0.227**	**-0.509**	**-0.717**	**-0.076**
	Nonomuraea	**-0.716**	**-0.698**	**-0.698**	**-0.698**	**0.039**	**-0.160**	**0.083**
	Ohtaekwangia	**0.776**	**0.793**	**0.793**	**0.793**	**0.089**	**0.623**	**-0.108**
	Ornatilinea	**0.641**	**0.652**	**0.652**	**0.652**	**0.029**	**0.407**	**0.174**
	Pedobacter	**-0.318**	**-0.227**	**-0.227**	**-0.227**	**-0.509**	**-0.717**	**-0.076**
	Planococcus	**-0.348**	**-0.274**	**-0.274**	**-0.274**	**-0.563**	**-0.739**	**-0.086**
	Porticoccus	**-0.318**	**-0.227**	**-0.227**	**-0.227**	**-0.509**	**-0.717**	**-0.076**
	Pseudomonas	**-0.458**	**-0.341**	**-0.341**	**-0.341**	**-0.519**	**-0.758**	**-0.007**
	Pseudoxanthomonas	**-0.495**	**-0.402**	**-0.402**	**-0.402**	**-0.045**	**-0.156**	**0.240**
	Rhodothermus	**0.643**	**0.656**	**0.656**	**0.656**	**0.026**	**0.404**	**0.144**
	Rummeliibacillus	**-0.042**	**-0.019**	**-0.019**	**-0.019**	**-0.093**	**-0.496**	**-0.304**
	Serpens	-0.755	-0.721	-0.721	**-0.721**	**-0.065**	-0.446	0.369
	Streptomyces	**-0.620**	**-0.602**	**-0.602**	**-0.602**	**-0.387**	**-0.127**	**0.107**
	Thermanaerothrix	**0.603**	**0.629**	**0.629**	**0.629**	**0.023**	**0.305**	**-0.117**
	Thermomarinilinea	**0.723**	**0.743**	**0.743**	**0.743**	**0.006**	0.496	0.204
Methanotroph	*Methylocaldum*	**0.837**	**0.882**	**0.882**	**0.882**	**-0.043**	0.649	0.075
	Methylococcus	**0.463**	**0.470**	**0.470**	**0.470**	**0.085**	**0.429**	**0.114**
	Methylobacter	-0.321	-0.467	-0.467	-0.467	0.445	**0.017**	**-0.328**
	Methylomicrobium	**-0.273**	**-0.393**	**-0.393**	**-0.393**	**-0.091**	**-0.248**	**-0.216**
	Methylosarcina	**0.041**	**-0.061**	**-0.061**	**-0.061**	**0.677**	**-0.251**	**-0.629**
	Methylomonas	**0.007**	**-0.037**	**-0.037**	**-0.037**	**0.806**	**-0.291**	**-0.531**
	Methylocystis	**0.142**	**0.023**	**0.023**	**0.023**	**0.739**	**-0.217**	**-0.335**
	Others	-0.533	**-0.602**	**-0.602**	-0.602	0.378	**-0.313**	**-0.099**

[a]Bold text indicates a significant difference ($p < 0.05$).

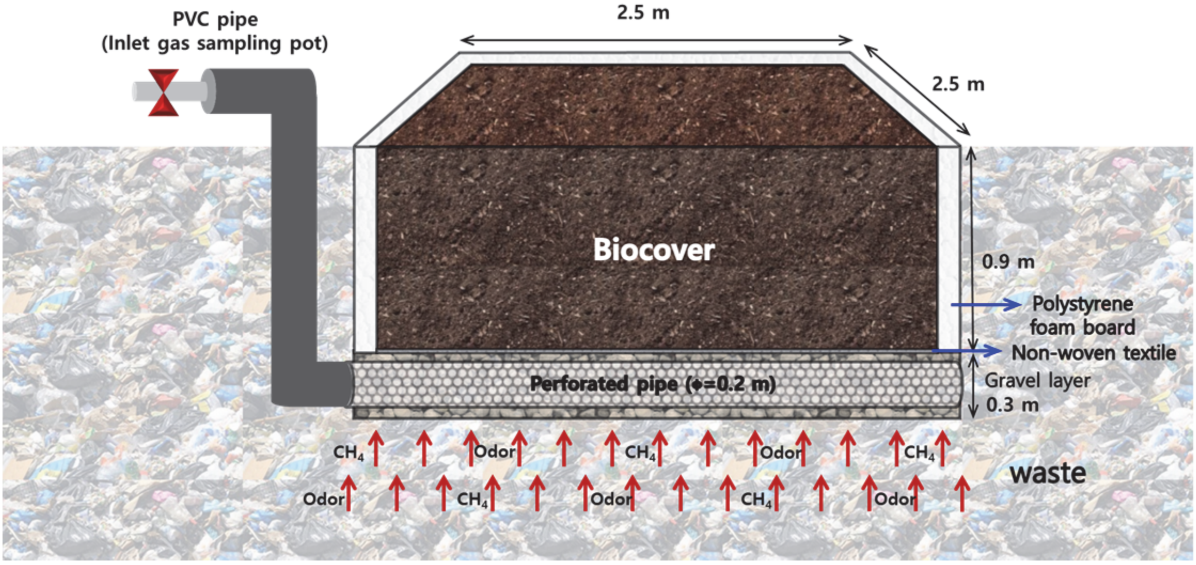

Fig. S1. Schematic diagram of the biocover constructed at a sanitary landfill in South Korea.
